# Surgical procedures and plasma exchange for ovarian teratoma-associated anti-N-methyl D-aspartate receptor encephalitis: a case report and review of literature

**DOI:** 10.3389/fonc.2023.1238087

**Published:** 2023-12-19

**Authors:** Yue Hu, Jianyuan Zhang, Peihai Zhang, Baozhi Sun, Hongli Zou, Lei Cheng

**Affiliations:** ^1^ Department of Gynecology and Obstetrics, Qilu Hospital (Qingdao), Cheeloo College of Medicine, Shandong University, Qingdao, China; ^2^ Department of Neurology, Qilu Hospital (Qingdao), Cheeloo College of Medicine, Shandong University, Qingdao, China

**Keywords:** ovarian teratoma, anti-N-methyl D-aspartate receptor encephalitis, plasma exchange, oophorectomy, adnexectomy, ovarian cystectomy, oophorocystectomy

## Abstract

We reported a case of ovarian teratoma-associated Anti-N-methyl-D-aspartate receptor (anti-NMDAR) encephalitis with recurrent epileptic seizures and disturbance of consciousness. Although surgical excision of the tumor remains the established standard of care, unlike other reported cases, the patient exhibited limited response to early oophorocystectomy, as well as IVIG and steroid therapy; however, a favorable response was observed with Plasma exchange (PE) initiated on postoperative day 12. Literature review revealed no definite recommended surgical extent for ovarian teratomas, and outstanding improvement in patients with anti-NMDAR encephalitis following PE. Our case raises the question regarding the optimal surgical extent for tumor resection, necessitating careful consideration when deciding between oophorectomy and adnexectomy as the preferred surgical procedure for anti-NMDAR encephalitis in female teens and adults. Furthermore, for refractory patients who fail to respond following tumor resection, PE can be performed early instead of immediately initiating second-line therapy.

## 1 Introduction

Ovarian teratomas are common ovarian germ cell tumors in women. Most of the teratomas are cystic and mature benign tumors, accounting for more than 95% of the ovarian teratomas (1). The tumors are mostly unilocular, with cavities filled with grease and hair, and sometimes teeth or bones are visible. The tumors are composed of multiple germ layers such as ectoderm, endoderm, and mesoderm, usually 2 to 3 germ layers, occasionally containing one single germ layer. Anti-N-methyl-D-aspartate receptor (anti-NMDAR) encephalitis is rare in patients with ovarian teratoma and is considered as the paraneoplastic neurological syndrome associated with teratoma (2). But not all teratomas trigger encephalitis, and not all anti-NMDAR encephalitis is associated with teratomas. Cases of anti-NMDAR encephalitis were first reported in 2005 (3). In 2007, Dalmau et al. proposed ovarian teratoma could induce release of anti-NMDAR antibodies that cause encephalitis, and named it anti-NMDAR encephalitis (4). The pathogenesis of teratoma-associated anti-NMDAR encephalitis remains unclear. It is postulated that the ectopic expression of N-methyl-D-aspartate receptors in nerve tissues of ovarian teratoma leads to production of anti-NMDAR antibodies, when normally the receptors are expressed in central nervous system and play an important role in synaptic transmission and regulation of synaptic plasticity (5). Anti-NMDAR antibodies cause selective cross-linking and internalization of the NMDAR (6), which lead to the occurrence of anti-NMDAR encephalitis.

As is considered an autoimmune disease, treatments of anti-NMDAR encephalitis focuse on reducing production of antibodies and receding immune response against neuronal autoantigens. First-line treatments include high-dose steroids, intravenous gamma globulin (IVIG), and plasma exchange (PE). In patients who do not have a response to first-line immunotherapy, second-line immunotherapy, such as rituximab or cyclophosphamide, may improve prognosis, but data on benefit of second-line immunotherapy are limited (7). Additionally, ovarian teratomas are the causative factor, thus ovarian teratoma resection or ovariectomy is an important procedure for management of ovarian teratoma-associated NMDAR encephalitis (8). In patients with tumors suitable for surgery, early removal of the tumor can accelerate improvement and reduce the chance of recurrence (9, 10), and Woo-jin Lee et al. emphasized surgery within 1 month of onset (11).

Here, we reported a case of ovarian teratoma-associated anti-NMDAR encephalitis, with admission of IVIG and steroid on day 2 after admission, and ovarian teratoma resection on day 6, but with no significant improvement. However, she gradually recovered from coma and seizures after initiation of PE from postoperative day 12. We would like this case could provide a reference for treatments of teratoma-associated anti-NMDAR encephalitis and bring further understanding of its pathogenesis.

## 2 Case presentation

An 11-year-old girl was admitted with mental and behavioral abnormalities for 11 days. At onset, she heard music playing continuously in her ears. For next 3 days, she was unable to sleep. Over next 2 days, she spoke loudly for no reason, kicked the table during meals, and ran around constantly. She could recall parts of the scene and told her parents she was mad. She went to local mental health center, where no abnormalities were found in her craniocerebral CT and electroencephalogram (EEG). She was treated with zopicron, olanzapine, clonazepam, and lorazepam. Her sleep improved, but she developed low fever (maximum 37.3° C), slurred speech, and involuntary jaw movement 3 days before admission. Her symptoms quickly aggravated with inability to communicate followed by incontinence. Upon admission, she was in a drowsy state, could not answer, partially acted as directed, and was not cooperative in physical examination. Muscle strength of lower limbs was grade 4. No pathological signs were elicited. At admission, auxiliary examination was completed. The examinations of blood routine, liver function, kidney function and blood lactic acid were all within normal limits, as well as the biochemical analysis of cerebrospinal fluid (CSF). CSF cytology revealed lymphocyte reaction. Influenza virus IgM antibodies were weakly positive. TORCH was negative. Female tumor markers (carbohydrate antigen 125, carbohydrate antigen 199, Human epididymis protein 4 and alpha fetal protein) were within normal values. Brain magnetic resonance imaging was unremarkable. Abdominal CT showed a mass in left adnexal area, with a high possibility of teratoma. EEG revealed widespread and persistent low-to-high-amplitude theta (4-7 Hz) waves and many medium-to-high-amplitude delta (2.5-3.5 Hz) waves. On day 2 of admission, she was lethargy and experienced convulsions and intermittent fevers. An empirical treatment was administered, which consisted of IVIG (22.5g/d), dexamethasone (10 mg/d), antiviral agents (acyclovir and oseltamivir), and ceftriaxone. On day 3, she lapsed into coma. On day 4, Anti-NMDAR antibodies were detected in both serum and CSF (serum titer: 1:100; CSF titer: 1:10), indicating the presence of ovarian teratoma-associated anti-NMDAR encephalitis. The administration of IVIG (22.5g/d for 5 days) and antiviral drugs were continued, while methylprednisolone (0.5g/d for 5 days) was substituted for dexamethasone, and concurrent antiepileptic therapy was administered. On day 6, laparoscopic ovarian tumor resection was performed. However, despite the administration of IVIG and steroids, as well as teratoma resection, the patient did not exhibit recovery from consciousness disturbance. Additionally, refractory convulsions occurred 6 days post-surgery. Twelve days after surgery, she received PE. Subsequently, she demonstrated signs of improvement, achieving a seizure-free state following administration of antiepileptic therapy. The titer of serum anti-NMDAR antibodies significantly decreased to 1:10. Following two courses of PE, she regained partial consciousness, intermittently responded to simple commands. After the fourth course of PE, she was conscious with normal dialogue and communication, the titer of CSF antibodies decreased to 1:1, and the serum antibody titer was negative. After a hospitalization of 42 days, she was discharged with an impressive Mini-Mental State Examination (MMSE) score of 28.

**Figure 1:**
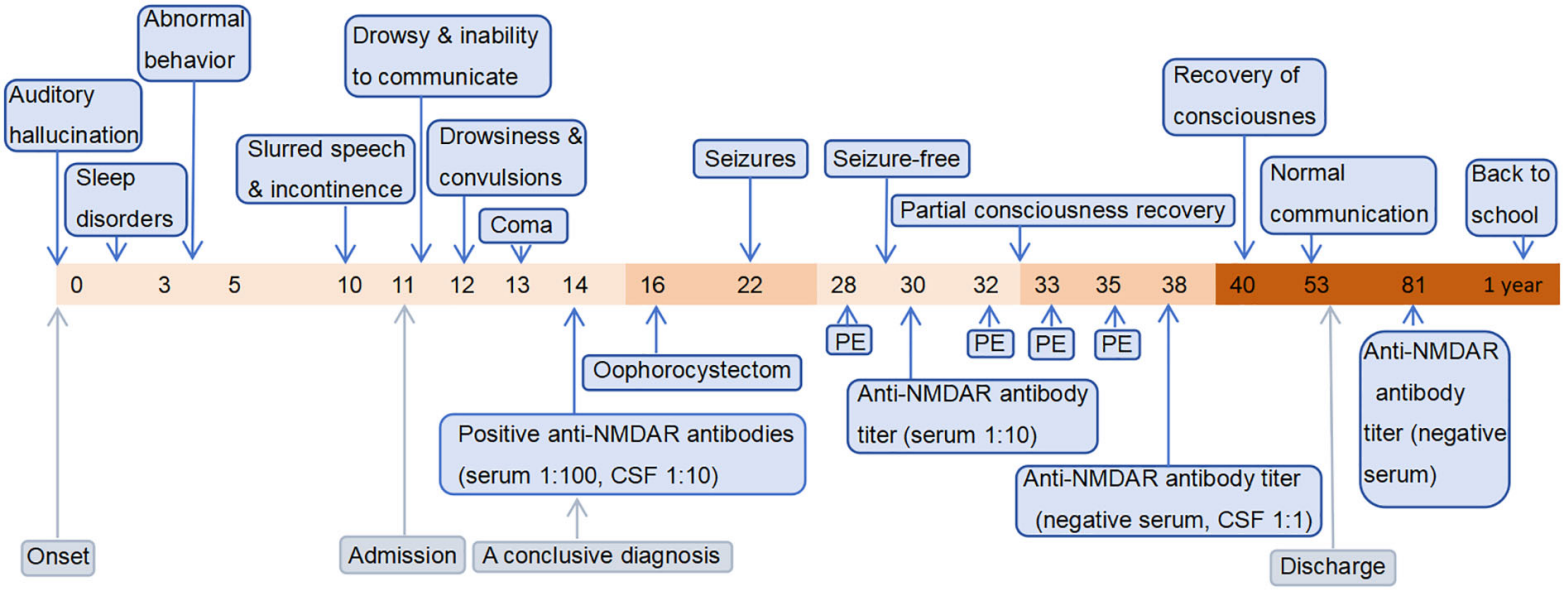
The timeline containing pertinent data from the episode of care. The horizontal axis represents time. The numerical values correspond to “days,” except for the final value which represents “1 year”.

She was given levetiracetam for maintenance therapy from discharge, and the dose of steroid was gradually reduced to withdrawal. 8 months after discharge, reexamination of the EEG revealed moderate abnormalities. Pelvic CT and gynecological ultrasound were unremarkable. 2 months before manuscript submission (about one year after diagnosis), she returned to school. She recovered well and was seizure-free but was still on maintenance therapy with levetiracetam and could not tolerate running in physical education classes. The timeline of her associated treatment course is illustrated in **Figure 1**.

**Figure 2:**
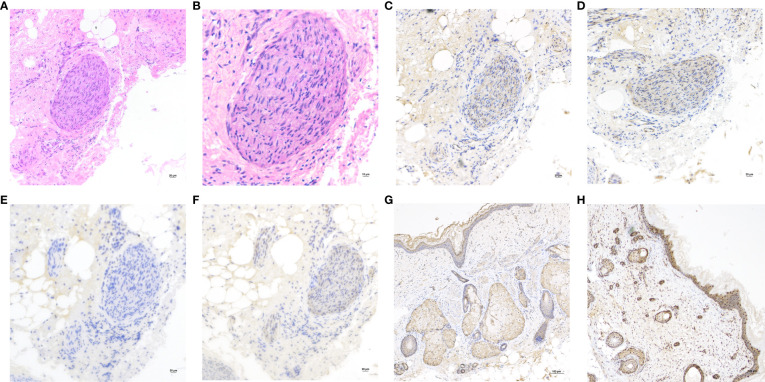
**(A)** Hematoxylin and eosin (H&E) staining of the nerve tissue in teratoma. Original magnification: ×200. **(B)** H&E, Original magnification: ×400. The nerve tissue showed reactivity for NMDAR1 **(C)**, NMDAR2B **(D)** and S100 **(F)**, but negative for MAP2 **(E)**. Original magnification: ×200. Immunohistochemistry showed that the sebaceous glands and squamous epithelium expressed NMDAR1 **(G)** and NMDAR2B **(H)**. Original magnification: ×100.

On the other hand, the pathological sections of the tumor tissue were examined by three senior gynecological pathologists, and nerve tissue was observed. Immunohistochemical staining (**Figures 2A–F**) of NMDAR1 and NMDAR2B in the nerve tissue was positive, MAP2 was negative, and S100 was positive. However, NMDAR1 and NMDAR2B were also positive in sebaceous glands and squamous epithelium (**Figures 2G, H**).

## 3 Discussion

In this case, the positive immunohistochemical staining of NMDA receptor in sebaceous gland and squamous epithelium of teratoma tissue was consistent with previous studies (12, 13), but the possibility of non-specific positive staining cannot be completely excluded. The pathogenesis of anti-NMDAR encephalitis is still unclear. There were studies suggested that all teratomas associated with anti-NMDAR encephalitis contained neural tissue that was positive for NMDA receptors (14, 15). However, it was also reported that NMDAR subunits were expressed in teratoma tissues of patients without encephalitis (16), as well as in normal ovaries (17), suggesting that teratoma was not a sufficient condition for disease, and explaining the onset of the disease in the absence of teratomas. Therefore, teratoma-associated NMDA receptor encephalitis may have other undetermined predisposing factors. This also explains why the patient’s symptoms did not improve after teratoma resection in this case.

Anti-NMDAR encephalitis is a treatable autoimmune disease characterized by prominent neuropsychiatric symptoms that predominantly affects young adults and children (18). About 26.9-44.2% of female patients are complicated with ovarian teratoma, which mainly occurs in young women and adolescents, and rarely in children (9–11, 19, 20). Tumor resection has long been regarded as an efficacious intervention for enhancing prognosis and reducing recurrence in teratoma-associated anti-NMDAR encephalitis; however, certain cases of ovarian teratoma-associated anti-NMDAR encephalitis fail to achieve remission or complete remission following teratoma resection. Study by Florace et al. showed a more frequently chance of full recovery occurred in patients who had a teratoma that was removed (5/8) than in those without a teratoma (4/23); and patients who did not improve after tumor resection or immunotherapy often have persistently high CSF antibody titers (20). Woo-jin Lee et al. emphasized patients with tumor resection within 1 month of onset had a better prognosis than those with removal of tumor within 3 months and 1 year (11). Jiang et al. reported that among 20 patients with teratoma-associated anti-NMDAR encephalitis, 17 patients with mature teratoma got clinical improvement after surgery, while the remaining 3 patients with immature teratoma had no improvement (13). These findings suggest that the histological subtype of teratoma plays a crucial role in guiding prognosis. However, despite the fact of the mature teratoma and a relatively low antibody titer of 1:10 in cerebrospinal fluid (CSF), our reported case did not demonstrate any benefits from early tumor resection.

In addition, there are few studies about the effects of different tumor resection extents on clinical treatment outcomes. It is unclear which of the three surgical modalities is more appropriate for patients with ovarian teratoma-associated anti-NMDAR encephalitis: oophorocystectomy, oophorectomy or adnexectomy. We noted that a study involving six patients with mature teratomas showed patients receiving oophorectomy did not have encephalitis recurrence during follow-up while 66.67% of those who underwent oophorocystectomy (two in three patients) had recurrent psychotic symptoms (21). However, it was difficulty to draw a conclusion due to the small patient numbers, and the author suggested the possible reason might be the removal of hidden teratoma components containing nerve tissue through oophorectomy (21). Furthermore, in the study by Jiang et al. mentioned above, of the 20 patients with surgical treatment, 6 patients (30.0%) received ovarian cystectomy, and the other 14 patients (70.0%) received affected unilateral adnexectomy (13); compared with surgical procedures, it was the benign or malignant nature of tumor that seems to be the prognostic factor. Nevertheless, Uchida et al. reported a case that failed to improve after teratoma resection, and was found a recurrent ovarian teratoma 6 months later, her clinical symptoms eventually improved after bilateral ovariectomy (22), which reminded us to be alert to the possibility of recurrence and residual of tumor resection.

Interestingly, though, Masghati et al. reported a case revealed gradual recovery following blind bilateral salpingo-oophorectomy, and ovarian teratoma was not identified at all by pathological examination (23). The case was unique and very rare, and effect of ovary surgery was recognized, yet a recommendation for oophorectomy for all the patients with anti-NMDAR encephalitis was of insufficient medicine evidence. And the case we reported which had no improvement after oophorocystectomy but recovered after PE highlighted an important role of PE in disease recovery and prompted us to question the necessary of oophorectomy and adnexectomy. According to the studies of immunohistochemical results (17), normal ovaries also expressed NMDAR, and ovariectomy and adnexectomy were not necessary for nulliparous women if PE could achieve the desired therapeutic effect and removed residual anti-NMDAR antibodies from the blood. Oophorectomy and adnexectomy are prudent therapeutic approaches, and for most female teens and young adults, consideration of fertility requirements and ovarian protection while avoiding residual tumor may be more critical in determining the surgical extent.

PE was considered effectively for anti-NMDAR encephalitis due to removal of circulating antibodies rapidly. Due to its invasive nature, high cost, and demanding technical requirements, PE is not typically considered as the first option in first-line treatment; moreover, it poses challenges in young patients who exhibit poor cooperation or prominent involuntary movements (24). Corticosteroids and IVIG was usually used before PE, yet there was no established recommendation for the order of administration. Early initiation of PE appears to be beneficial (25–27). A review of 71 articles with 242 subjects confirmed a trend toward a better outcome when PE was administered early, and when given with steroids (27). Meanwhile, there were studies showed patients who received IVIG after PE did better than those who received IVIG before PE, and suggested PE should be considered prior to IVIG for rapid improvement and a better outcome (25, 26). And PE might rapidly improve the clinical manifestations in patients with severe refractory anti-N-methyl-D-aspartate (anti-NMDA) receptor encephalitis (28, 29). We report a case diagnosed of teratoma-associated anti-NMDAR encephalitis that failed to benefit from steroid and IVIG treatments, as well as teratoma resection, but responded well to PE. Her initiation of steroid and IVIG was approximately 12 days after the onset of symptoms, and teratoma removal was performed 16 days after disease onset, PE was initiated from day 32 after onset for coma and refractory convulsions. Although a better clinical outcome due to other therapies cannot be excluded completely, and a causal relationship between PE and her recover cannot be established, the strong temporal relation of improvement to PE demonstrated a possibility that initiation of PE could be beneficial.

The factors underlying the lack of resolution of anti-NMDAR encephalitis after teratoma resection remain poorly understood (**Table 1**). None of the potential associated factors for non-response reported in previous literature were observed in this case. We propose that despite the removal of the teratoma, which potentially acted as a causative factor, there remains a significant presence of antibodies in both the patient’s bloodstream and cerebrospinal fluid. Although steroid and IVIG can alleviate symptoms by exerting anti-inflammatory effects and modulating the immune system, they are unable to completely eradicate all antibodies (30). PE provides a direct approach to eliminate pathogenic antibodies along with other immune components from circulation, thereby rapidly improving symptoms. Additionally, we postulated that the presence of NMDA receptors in normal ovarian tissue may lead to the generation of anti-NMDAR antibodies even after teratoma removal, and this process could potentially be terminated by the combination therapy with steroid, IVIG, and PE.

**Table 1:** Review of reported cases of non-response to surgical treatment.

Year	authors	Number of cases	Surgical extent	Possible reasons for nonresponse to surgical treatment
2009	Florance et al.	8	Tumor removal (extent unspecified)	Persistently high CSF antibody titers (although serum titers may significantly decrease following tumor resection or immunotherapy)
2018	Uchida et al.	1	Ovarian cystectomy	Recurrence of the ovarian teratoma
2020	Lee et al.	31	Teratoma removal (extent unspecified)	Delayed removal of a teratoma (symptom onset to removal interval of 1 month or more)
2020	Yu et al.	6	Three patients underwent unilateral oophorocystectomy and the other three underwent unilateral oophorectomy	Possible residual of occult teratoma component with neural tissue
2021	Jiang et al.	20	Six patients received ovarian cystectomy and fourteen received affected unilateral adnexectomy	The central hypoventilation symptom and the immature pathological type of ovarian teratoma

We also noted that the patient was weakly positive for influenza virus IgM antibodies, so empirical antiviral therapy (acyclovir and oseltamivir), IVIG and dexamethasone were given after admission, but then the patient was diagnosed as teratoma-associated anti-NMDAR encephalitis according to the patient’s condition and auxiliary examination. Virus infection is one of the causes of anti-NMDAR encephalitis. prodromal infection symptoms or events occur in some patients with anti-NMDAR encephalitis. It has been reported that viral encephalitis can be followed by anti-NMDAR encephalitis (31). Patients with viral encephalitis often have high fever with acute onset, while anti-NMDA receptor encephalitis has normal body temperature or intermittent fever with subacute onset. Severe viral encephalitis can cause disturbance of consciousness or even coma, but psychiatric symptoms are relatively rare. There is therefore insufficient evidence of viral encephalitis in the present case. Nevertheless, we will also pay attention to whether viral infection affects the therapeutic response in teratoma-associated anti-NMDAR encephalitis in the future.

In summary, this case illustrated some aspects of therapy strategies of teratoma-associated anti-NMDAR encephalitis. There are two noteworthy points derived from this case: 1) The consideration of ovarian protection and fertility preservation is crucial, with oophorocystectomy being preferred over oophorectomy or adnexectomy in younger patients for mature teratoma. It would be unwise to remove the ovaries of all patients since NMDAR expression may also exist in normal ovarian tissue. Early implementation of PE can serve as an important treatment guarantee for patients with ovarian preservation. Therefore, for unilateral teratoma, oophorocystectomy is recommended for adolescents and women of childbearing age, ipsilateral adnexectomy for women older than 45 years who do not desire fertility preservation, and bilateral adnexectomy for postmenopausal women. In the case of bilateral teratomas, bilateral ovarian tumor resection is feasible prior to menopause, while bilateral adnexectomy is feasible after menopause. It is essential to provide personalized treatment options following a comprehensive consideration of the patient’s condition and wishes. 2) Despite the absence of symptom amelioration following IVIG and steroid, as well as early teratoma removal, PE may still be considered the preferred initial treatment option rather than immediate initiation of second-line interventions.

## 4 Conclusions

In conclusion, we present a case of ovarian teratoma-associated anti-NMDAR encephalitis that remained unresolved despite tumor resection, hormone therapy, and IVIG treatment. However, the patient’s condition improved significantly following PE intervention. Surgical removal of the tumor is essential in cases of teratoma-associated anti-NMDAR encephalitis; nevertheless, gynecologists should exercise caution when selecting surgical extent and consider the potential benefits of early implementation of PE for patients with refractory symptoms. Furthermore, while this case report provides some scientific basis, its evidence remains limited. Future research should continue to focus on optimizing treatment strategies and identifying factors influencing treatment response.

## Data availability statement

The original contributions presented in the study are included in the article/supplementary material. Further inquiries can be directed to the corresponding author.

## Ethics statement

Written informed consent was obtained from the minor(s)’ legal guardian/next of kin for the publication of any potentially identifiable images or data included in this article.

## Author contributions

YH wrote the manuscript. YH and JZ managed the patient. PZ supervised the operation. BS and HZ gathered the clinical information. LC performed the surgical procedure, reviewed and edited the manuscript. All authors contributed to the article and approved the submitted version.
